# P-114. Nasal-Spray Bacillus Spore Probiotics Prevent Respiratory Infections in Preschoolers: A Community RCT

**DOI:** 10.1093/ofid/ofaf695.342

**Published:** 2026-01-11

**Authors:** Anh Tran Pham, Thu Thi Hoai Nguyen, Huyen Thi Bui, Anh Thi My Bui, Hung Thanh Phung, Anh Quynh Pham, Huong Thi Le, Hai Hoang Tran, Viet Duc Le, Anh Hoa Nguyen, Tung Dinh Pham, Nga Thi Tuyet Nguyen, Anh Thi Van Nguyen

**Affiliations:** Hanoi Medical University, Hanoi, Ha Noi, Vietnam; Hanoi Medical University, Hanoi, Ha Noi, Vietnam; ANABIO R&D Ltd., Hanoi, Ha Noi, Vietnam; Hanoi Medical University, Hanoi, Ha Noi, Vietnam; Hanoi Medical University, Hanoi, Ha Noi, Vietnam; Hanoi University of Public Health, Hanoi, Ha Noi, Vietnam; Hanoi Medical University, Hanoi, Ha Noi, Vietnam; Spobiotic Research Center, ANABIO R&D Ltd. Company, Hanoi, Ha Noi, Vietnam; Spobiotic Research Center, ANABIO R&D Ltd. Company, Hanoi, Ha Noi, Vietnam; LiveSpo Pharma Ltd., Hanoi, Ha Noi, Vietnam; VNU University of Science, Vietnam National University_Hanoi, Hanoi, Ha Noi, Vietnam; Universitätsklinikum Tübingen, Wilhelmstrasse, Thuringen, Germany; Spobiotic Research Center, ANABIO R&D Ltd. Company, Hanoi, Ha Noi, Vietnam

## Abstract

**Background:**

Respiratory tract infections (RTIs) are a leading cause of pediatric morbidity and excessive medication use, contributing to antimicrobial resistance. Current preventive strategies remain limited in long-term effectiveness.

**Figure 1:**
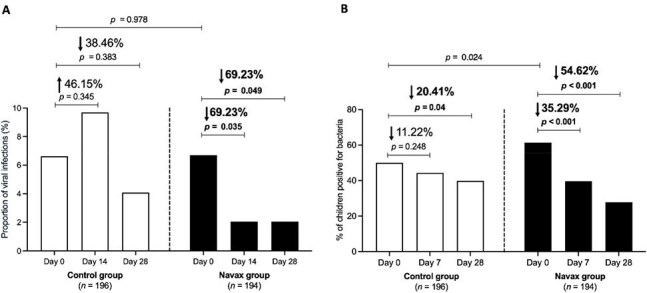
Incidence of viral (A) and bacterial (B) infections in the Control group (white bars) and Navax group (black bars) across all participants at days 0, 14, and 28.

**Methods:**

Here, we evaluated the efficacy of nasal-spraying *Bacillus* spore probiotics in reducing RTI incidence and medication use among preschool children. In a randomized, double-blind, controlled trial, preschool children aged 2–5 in Hanoi, Vietnam received daily nasal sprays for 28 days during the winter-spring transition. The intervention (Navax) group received LiveSpo NAVAX (>1 billion CFU/mL of *B. subtilis* ANA4 and *B. clausii* ANA39), while the Control group received physiological saline. Clinical symptoms, medication usage, viral/bacterial infections, and nasal microbiota were assessed at days 0, 14, and 28.

**Results:**

Compared to control, the Navax group showed significant reductions in RTI symptoms, including runny nose (−42.4%), tonsillitis (−83.3%), tonsillitis-adenoiditis (−61.7% at day 14; −36.2% at day 28), hoarseness (−66.7%), and sore throat (−73.9%). Medication use (antibiotics, anti-inflammatories, cough suppressants, expectorants, and antihistamines) decreased by 27–75% (*p* < 0.05). Viral infection incidence dropped by 69.2%, with a 92.3% reduction in rhinovirus cases, and risk of acquiring new viral infections was reduced by 5–6-fold. Bacterial co-infections also decreased: *M. catarrhalis* (−50%), *H. influenzae* (−67.6%), and *S. pneumoniae* (−40.5%). Nasal microbiota analysis revealed increased abundance of beneficial genera (*Brevibacillus*, *Bacillus*, *Flavobacterium*) and decreased pathogenic taxa (*Moraxella*, *Dolosigranulum*, *Haemophilus*).

**Conclusion:**

This study provides the first clinical evidence that daily nasal-spraying *Bacillus* spores offer a safe, microbiome-friendly, and broad-spectrum strategy to reduce respiratory infections, lower medication use, and prevent both viral and bacterial transmission in young children.

**Disclosures:**

Anh Hoa Nguyen, PhD. in Life Sciences, LiveSpo Pharma Ltd. Company: Board Member Anh Thi Van Nguyen, PhD, LiveSpo Pharma Ltd. Company: Advisor/Consultant

